# Persistence of Diffusion Capacity Impairment and Its Relationship with Dyspnea 12 Months after Hospitalization for COVID-19

**DOI:** 10.3390/jcm13051234

**Published:** 2024-02-21

**Authors:** Alice Kang, Binaya Regmi, Christian Cornelissen, Judith Smith, Ayham Daher, Michael Dreher, Jens Spiesshoefer

**Affiliations:** 1 Department of Pneumology and Intensive Care Medicine, University Hospital RWTH Aachen, 52074 Aachen, Germany; akang@ukaachen.de (A.K.);; 2 Interdisciplinary Health Science Center, Scuola Superiore Sant’Anna, 56127 Pisa, Italy

**Keywords:** COVID-19, coronavirus, diffusion capacity, pulmonary function, dyspnea

## Abstract

**Background**: Dyspnea is a common persistent symptom after acute coronavirus disease 2019 illness (COVID-19). One potential explanation for post-COVID-19 dyspnea is a reduction in diffusion capacity. This longitudinal study investigated diffusion capacity and its relationship with dyspnea on exertion in individuals previously hospitalized with COVID-19. **Methods**: Eligible participants had been hospitalized for the treatment of acute COVID-19 and were assessed at 6 weeks, 6 months, and 12 months after discharge. Pulmonary function testing, diffusion capacity of carbon monoxide (DLCO), blood gas analysis and the level of dyspnea (Borg scale; before and after a 6 min walk test [6 MWT]) were performed. Participants were divided into subgroups based on the presence or absence of dyspnea during the 6 MWT at 12 months after hospitalization. **Results**: Seventy-two participants (twenty-two female, mean age 59.8 ± 13.5 years) were included. At 12 months after discharge, 41/72 participants (57%) had DLCO below the lower limit of normal and 56/72 (78%) had DLCO < 80% of the predicted value. Individuals with exertional dyspnea had significantly lower DLCO than those without exertional dyspnea (*p* = 0.001). In participants with DLCO data being available at three timepoints over 12 months (baseline, 6 months, and 12 months) after discharge (*n* = 25), DLCO improved between 6 weeks and 6 months after hospital discharge, but not thereafter (*p* = 0.017). **Conclusions**: About 2/3 of the post-COVID individuals in this study had impaired diffusion capacity at 12 months after hospital discharge. There was an association between persisting dyspnea on exertion and significantly reduced DLCO. Impaired diffusion capacity improved over the first 6 months after hospitalization but not thereafter.

## 1. Introduction

More than three years have passed since the start of the coronavirus disease identified in 2019 (COVID-19) pandemic. This means that there are very large numbers of individuals who have recovered from the acute phase of disease. While most have no ongoing impacts, a small but relevant proportion of individuals experience persistent symptoms and abnormalities [1,2,3,4]. The terms used for these persistent issues after recovery from acute COVID-19 illness include long COVID and post-COVID syndrome. 

The pathophysiological mechanisms underlying the persistent symptoms are not fully understood. Functional abnormalities of survivors of COVID-19 pneumonia can persist and are associated with initial disease severity [5]. A recovery of exercise capacity impairment is reported by three to six months after acute illness in most cases [6]. In a large prospective study, an association was found between DLCO and mMRC and CAT score at three and six months after COVID-19. This depicts to some extent the inconsistency in the current literature [7]. Nevertheless, additional research is needed to help fully understand the causes of unexplained exertional dyspnea in individuals with long COVID.

Diffusion capacity of the lungs for carbon monoxide (DLCO) has been shown to be reduced after hospitalization for acute COVID-19 [1,8]. However, there is a discrepancy between the findings of pulmonary function tests and patient-reported outcome measures, whereby improvements in pulmonary function between three and six months after COVID-19 onset were not matched by improvements in patient-reported outcomes [9]. In another study, although some patients showed improvements in DLCO, dyspnea, and exercise capacity in the year after recovery from severe COVID-19, nearly a quarter had persistent impairments [10]. 

This study evaluated the DLCO and determined its relationship with exertional dyspnea in the year after hospital discharge in individuals previously hospitalized for acute COVID-19 illness. This longitudinal approach allows the evaluation of changes in impaired DLCO over time, which provides insights into the sequelae of COVID-19, long COVID syndrome, and any expected improvements. 

## 2. Materials and Methods

### 2.1. Study Design and Participants

This prospective study (German Clinical Trials Register, DRKS00027106) was conducted at the University Hospital RWTH Aachen, Germany. The study protocol of the COVAS registry was approved by the local Ethics Committee University Hospital RWTH Aachen (approval number: EK 080/20; approval date: 27 March 2020) and all participants were approached after providing written informed consent during their hospitalization. All investigations were performed in accordance with the ethical standards laid down in the declaration of Helsinki in the latest revision. 

Inclusion criteria were age > 18 years and hospitalization for the treatment of COVID-19 between February 2020 and November 2021. The presence of severe acute respiratory syndrome coronavirus type 2 (SARS-CoV-2) infection was confirmed by reverse transcriptase polymerase chain reaction analysis of a respiratory tract sample. Exclusion criteria were pre-existing reasons for the reduced DLCO, including chronic obstructive pulmonary disease (COPD), chronic heart failure, or pulmonary fibrosis. According to the COVID-19 Treatment Guidelines, severe illness was defined as SpO_2_ < 94% on room air at sea level, a ratio of arterial partial pressure of oxygen to fraction of inspired oxygen (PaO_2_/FiO_2_) < 300 mmHg, a respiratory rate > 30 breaths/min, or lung infiltrates > 50%; and critical illness was defined as respiratory failure, septic shock, and/or multiple organ dysfunction [11]. A computer tomography (CT) scan of the chest was performed on all patients and evaluated using the COVRADS score [12].

Pharmacological treatments were given in accordance with the available evidence at the time, including corticosteroids in patients requiring supplemental oxygen (i.e., all participants in our study). Evidence for the use of other treatments, including monoclonal antibodies, antivirals, or other agents, became available over time and was implemented accordingly. Individuals with prolonged weaning from mechanical ventilation were referred for pulmonary rehabilitation.

### 2.2. Routine Follow-Up

Data on the use of mechanical ventilation and oxygen therapy details were retrieved from hospital medical records. Follow-up assessments were scheduled at approximately 6 weeks, 6 months, and 12 months after hospital discharge. 

With support from a trained study team, participants answered clinical questionnaires to determine dyspnea (Borg dyspnea scale [13]). Whole-body plethysmography (MasterLab, Viasys, Hoechberg, Germany) before and after bronchodilatation was performed according to current guidelines [14,15] (DLCO was determined after bronchodilation only and was corrected for hemoglobin values as per standard clinical routine). Samples for capillary blood gas analysis were taken from the arterialized earlobe of all participants while breathing room air without supplemental oxygen (ABL 800 flex, Radiometer, Copenhagen, Denmark). Borg dyspnea scale scores were also determined before and after a 6 min walk test without supplemental oxygen [13,14]. The 6 min walk tests were carried out according to guidelines. The 6 min walk test (6 MWT) is a commonly used test for the objective assessment of functional exercise capacity for the management of patients with moderate-to-severe pulmonary disease. The patient is asked to walk as far as possible along a 30 m corridor for a period of 6 min with the primary outcome measure being the 6 min walk distance (6 MWD) measured in meters [16]. Participants were then classified based on the presence or absence of dyspnea at 12 months after hospital discharge: no dyspnea (a Borg dyspnea scale score of 0–2) or dyspnea (a Borg dyspnea scale score of 3–10). The lower limit of normal was calculated using the ERS calculator [17].

### 2.3. Outcomes

The primary endpoint was the correlation between the DLCO and the presence of exertional dyspnea at 12 months. Changes in the DLCO over the period from 6 weeks to 12 months after hospital discharge were also assessed where possible.

### 2.4. Statistical Analysis

Statistical analysis was performed using Microsoft Excel and Sigma PlotTM software (Version 13.0, Systat, Erkrath, Germany), IBM Corp. Released 2021. IBM SPSS Statistics for Windows, Version 28.0. IBM Corp, Armonk, NY, USA and GraphPad Prism, prism 10 for macOS (Version 10.0.2 (171), 31 July 2023), Boston, MA 02110, USA. A *p*-value ≤ 0.05 was considered statistically significant in all analyses. A Shapiro–Wilk-Test was performed on all parameters before proceeding to the respective tests to demonstrate the normality of the data presented as mean with SD. Fisher’s exact *t*-test was used for between-group comparisons, as appropriate, after the application of Levene’s Test for homogeneity of variance in the form of an F-test. Comparisons between patient subgroups based on the presence or absence of exertional dyspnea at 12 months after hospital discharge were performed using a *t*-test for independent samples. Analysis of variance (ANOVA) was used to evaluate changes over time. Bonferroni post hoc analysis was performed on individuals with data available at 6 weeks, 6 months, and 12 months after hospital discharge after testing for normal distribution and homogeneity of variance. Pearson correlation and R/R-squared were determined to investigate factors associated with exertional dyspnea at 12 months after hospital discharge.

## 3. Results

### 3.1. Study Participants

Of 276 individuals with COVID-19 who required invasive mechanical ventilation in the intensive care unit (*n* = 110) or received supplemental oxygen only (*n* = 166) and were seen for follow-up in the outpatient clinic at 12 months after hospital discharge, 72 (mean age 59.8 ± 13.5 years) fulfilled all inclusion criteria, had complete data on DLCO and Borg scale ratings at rest and after exercise at 12 months, and were still alive, and were therefore enrolled in this study; of these, 30 had exertional dyspnea at the 12-month follow-up and 42 did not (Table 1). Twenty-one individuals met the Berlin definition [18] for severe acute respiratory distress syndrome during the initial hospitalization and received invasive mechanical ventilation. A subgroup of 25 individuals had DLCO data from assessments at 6 weeks, 6 months, and 12 months after hospital discharge and were included in the analysis of longitudinal changes in DLCO. 

### 3.2. Comparisons between Individuals with and Those without Dyspnea

**Figure 1:**
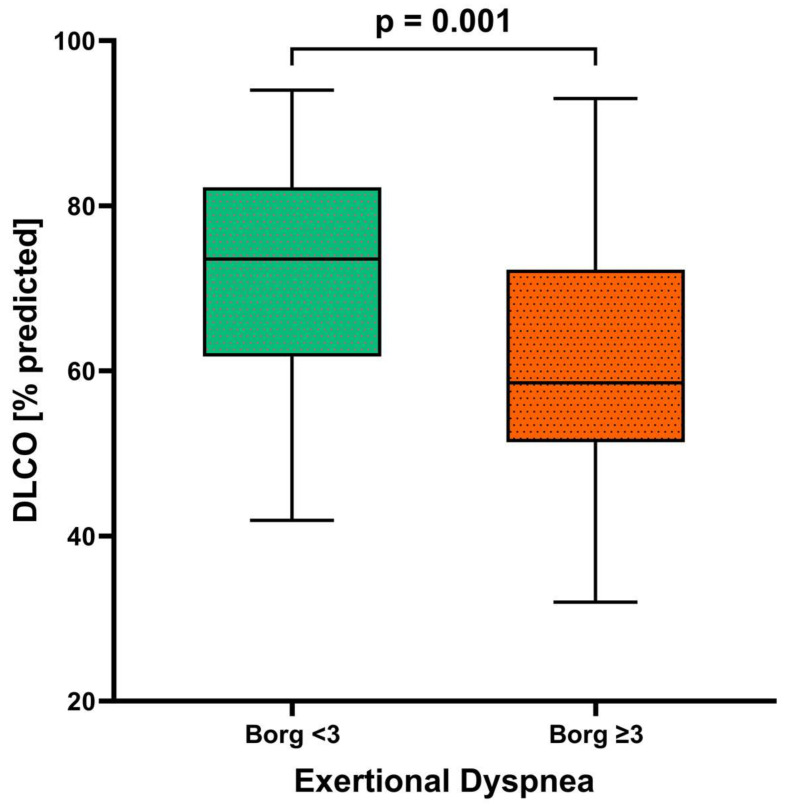
Diffusion capacity (DLCO) in patients with (*n* = 30) and without (*n* = 42) dyspnea after 12 months (*n* = 72). The *p*-value was determined with a *t*-test for independent samples.

**Figure 2:**
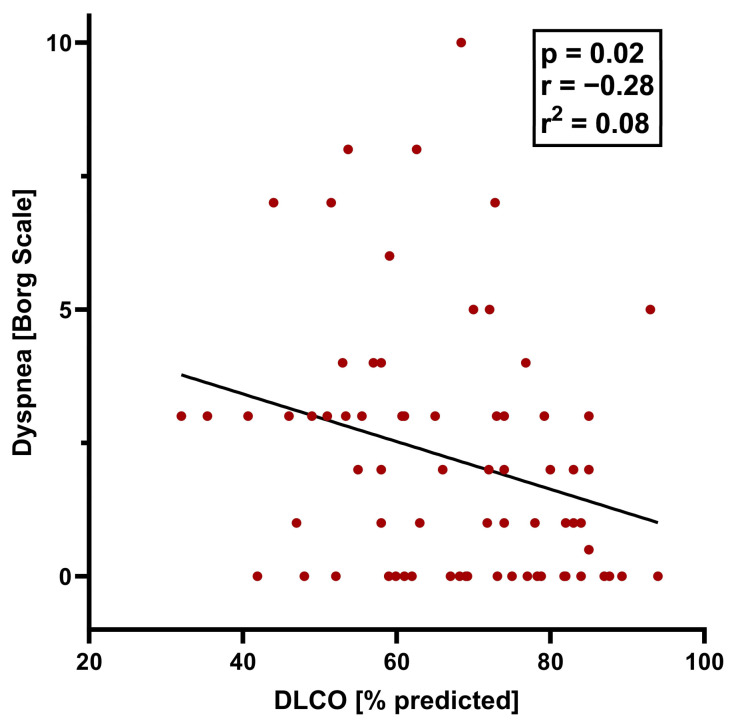
Pearson correlation between DLCO and dyspnea after exercise (Borg scale score > 3) 12 months after hospital discharge.

Individuals with dyspnea at the 12-month follow-up differed significantly from those without dyspnea in a variety of lung function parameters, including a lower total lung capacity (TLC), vital capacity (VC), and forced expiratory volume in one second (FEV_1_), and higher residual volume (RV)/TLC ratio and effective resistance. However, the FEV_1_/FVC ratio did not differ significantly between the dyspnea and no dyspnea groups (Table 1). The DLCO and DLCO/alveolar volume ratio were significantly lower in individuals with versus without exertional dyspnea, and the proportion with DLCO below the lower limit of normal was significantly higher (Table 1; Figure 1). In all participants, exercise was associated with a significant worsening of dyspnea, although the Borg scale score remained significantly higher in those with versus without dyspnea (Table 1, Figure 1). On a formal statistical level, the correlation between the DLCO and exertional dyspnea was weak (R^2^ = 0.07571, *p* = 0.021) (Figure 2). A higher body mass index was associated with dyspnea on exertion, as were lung diseases other than COPD, asthma, obstructive sleep apnea, and chronic kidney disease; smoking status was not significantly associated with exertional dyspnea (although this study excluded patients with COPD). 

Even though women had significantly smaller TLC [L], VC [L], FEV_1_ [L], DLCO [mmol/(min*kPa)], and DLCO/VA [mmol/(min*kPa*L)] than men, there was no statistically significant difference between one-year outcomes in terms of dyspnea or exertional dyspnea. Although there was no statistically significant difference between men and women with critical or severe disease, the percentage of men was higher.

### 3.3. Changes in Lung Function and Diffusion Capacity over Time

**Figure 3:**
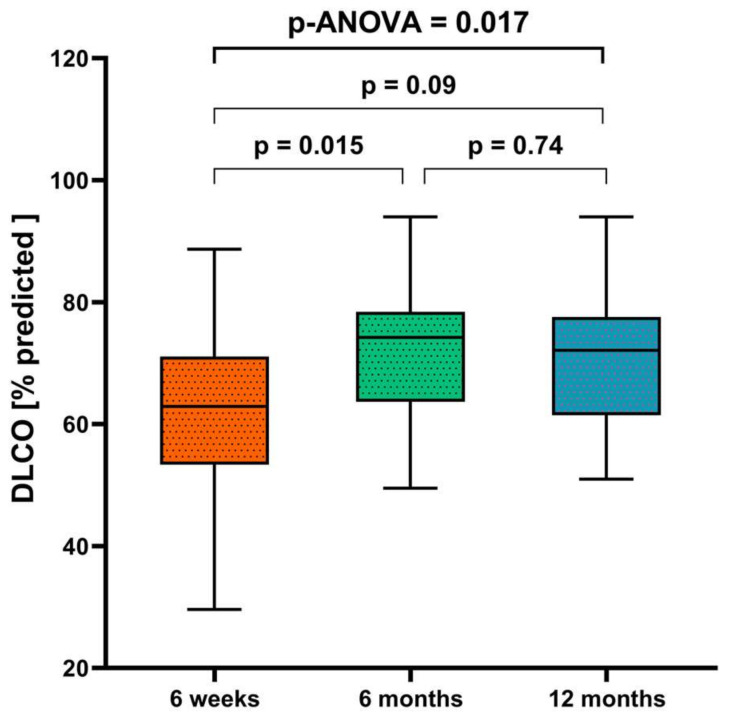
Diffusion capacity at 6 weeks, 6 months, and 12 months after hospital discharge (*n* = 25). The *p*-value was determined using analysis of variance (ANOVA).

At 12 months after discharge, 41/72 patients (57%) had DLCO below the lower limit of normal, 56/72 (78%) had DLCO below 80% of the predicted value, 23/72 patients (32%) had DLCO/VA below the lower limit of normal, and 28/72 (39%) had DLCO/VA below 80% of the predicted value (Table 1). There was no evidence of a restrictive lung function pattern (Table 2). The absolute values for TLC and VC differed significantly between males and females, but the % of predicted values of these parameters were similar in both men and women (Table 1). In a subset of participants with DLCO data at multiple timepoints (*n* = 25), the percent of predicted DLCO increased significantly from 6 weeks to 6 months, but did not significantly change from 6 to 12 months (Table 2, Figure 3); a similar pattern of changes was seen for RV.

## 4. Discussion

The results of this study suggest that individuals hospitalized for the treatment of moderate to severe COVID-19 have impaired diffusion capacity, regardless of the use of mechanical ventilation during hospitalization, and that this may recover to some extent over the first 6 months after hospitalization, but not thereafter. This reduced diffusion capacity is associated with persistent dyspnea symptoms at 12 months after hospital discharge.

Lung abnormalities, such as remodeling, vascular changes, and micro-thrombosis, are common after acute COVID-19 illness [9,19,20,21,22,23]. Our findings are consistent with a previous COVID-19 follow-up study conducted by a Chinese study group, that excluded individuals treated with mechanical ventilation and those who had diabetes, cardiovascular diseases, hypertension, nicotine abuse and/or pulmonary diseases [10]. The most common residual pulmonary function abnormality after 12 months was reduction in gas transfer, as measured by DLCO [10]. We also identified an impaired recovery of diffusion capacity over 12 months after hospitalization for COVID-19 as a correlate for persistent dyspnea, even in the absence of any other obvious reason for dyspnea/impaired diffusion capacity. 

The impairment of DLCO after hospital discharge could be a consequence of interstitial abnormalities or pulmonary vascular abnormalities caused by COVID-19. This is supported by data from a Canadian study group, which showed that radiological impairment on chest CT was associated with the duration of oxygen supplementation [9]. Another potential underlying cause of lung impairment after acute COVID-19 illness is the presence of immuno-thrombosis with micro-thrombosis and angiogenesis/vessel enlargement seen on dual energy CT in these individuals [23]. Ackermann et al. investigated post-mortem morphologic and molecular lung features in individuals who had had COVID-19 and found that micro-thrombi, lymphocytic inflammation, and intussusceptive angiogenesis were the most distinctive findings, even in the absence of mechanical ventilation [19].

Although data were only available from a subset of participants, this study is one of the first to perform a longitudinal assessment of diffusion capacity of acute COVID-19 illness. In a previous study, a Berlin-based group reported that pulmonary restriction and reduced DLCO correlated with clinical disease severity during acute COVID-19, that there were significant improvements in lung function (including DLCO) during the 12 months after acute illness, and that there was an association between lung involvement during acute illness and pulmonary restriction and impaired DLCO during follow-up [24]. Our data add to this by showing that diffusion capacity did not differ between those with or without mechanical ventilation during hospitalization and that the persistence of diffusion capacity impairment correlated with otherwise unexplained dyspnea at 12 months after hospitalization for COVID-19.

This study provides useful insight into the course of lung function, especially diffusion capacity, in the year after discharge from COVID-19 illness-related hospitalization. According to our data, the course of DLCO in the follow-up explains the residual impairment and associated dyspnea in long COVID patients after the subacute phase and recovery. Those patients who suffer from dyspnea after 12 months have a significant reduction in TLC volume and VC volume as an expression of a restrictive ventilation disorder. Sex differences can be observed as expected in TLC, VC, and RV as absolute volume. Even FEV_1_ and DLCO as absolute values were different between men and women. However, this is not surprising as body constitution and its proportion to the lungs should be different. Therefore, absolute values but not the % of predicted values differ according to the presence of exertional dyspnea when it comes to lung volumes. It is remarkable that there is a higher percentage of men than women both with severe and critical disease, but without a statistically significant difference. In the literature, focusing between sex differences in COVID-19 mortality indicates that men are more prone to die of SARS-CoV-2 infection than women [25,26]. Estrogen interaction with the renin–angiotensin–aldosterone system, one of the most critical pathways in COVID-19 infectivity, and the modulation of the vasomotor homeostasis are discussed. Testosterone, on the contrary, enhances the levels of the two most critical molecules, angiotensin-converting enzyme 2 (ACE2) and the transmembrane protease serine-type 2 (TMPRSS2), transcriptionally and post-transcriptionally, thereby increasing viral load and delaying viral clearance in men as compared to women [26]. 

Our study also has some limitations. Firstly, the total sample size was modest, and the subgroup of participants with data available prior to the 12 months assessment was small. Therefore, the findings should be considered as hypothesis-generating only and interpreted with caution. Nevertheless, we identify another potential contributor (diffusion capacity impairment) to otherwise unexplained dyspnea in individuals with long COVID, and highlight that diffusion capacity might be expected to improve from 6 weeks to 6 months after hospital discharge but not from 6 months to 12 months. Due to the nature of the current study, these findings need to be confirmed in larger prospective studies. Secondly, the availability of pulmonary function test data from before SARS-CoV-2 infection would have allowed a better understanding of changes over time and provided a more complete picture of the impact of COVID-19 illness on diffusion capacity. Nevertheless, all participants were able to perform all activities of daily living before becoming ill with COVID-19, and only reported issues with these tasks after recovering from acute COVID-19 illness.

## 5. Conclusions

In summary, one of the determinants of dyspnea is reduced diffusion capacity. This study showed that the persistence of diffusion capacity impairment over 12 months after hospitalization for severe COVID-19 is associated with ongoing dyspnea in these individuals. In addition, the current data showed that the recovery of reduced diffusion capacity is possible within the first 6 months after the acute phase of COVID-19, but not thereafter. The impaired diffusion capacity of the lungs plays a role in long-term respiratory outcome but is not the only determinant for dyspnea. Further studies are needed to explain persistent dyspnea in long COVID patients. 

## Figures and Tables

**Table 1:** Demographic and clinical characteristics of study participants, overall and in subgroups, based on the presence or absence of dyspnea 12 months after hospital discharge.

	All Participants				Dyspnea				No Dyspnea				Dyspnea vs. No Dyspnea
	Overall (*n* = 72)	Females (*n* = 22)	Males (*n* = 50)	*p_mw*-Value	Overall (*n* = 30)	Females (*n* = 12)	Males (*n* = 18)	*p_D* *mw*-Value	Overall (*n* = 42)	Females (*n* = 10)	Males (*n* = 32)	*p_NDmw*-Value	*p*-Value
**Characteristics**													
Age, years	59.8 ± 13.5	60.1 ± 11.1	59.3 ± 14.4	0.67	62.0 ± 16.0	62.3 ± 10.8	61.8 ± 19.0	0.95	58.2 ± 11.2	59.1 ± 11.7	57.9 ± 11.2	0.77	0.24
Height, m	1.73 ± 0.1	1.64 ± 0.5	1.77 ± 0.1	**<0.001**	1.69 ± 0.1	1.62 ± 0	1.74 ± 0.1	**<0.001**	1.76 ± 0.1	1.67 ± 0.1	1.79 ± 0.1	**<0.001**	**0.001**
Body weight, kg	88.4 ± 15.6	82.8 ± 18.2	90.9 ± 13.8	**0.041**	89.8 ± 19.1	83.2 ± 19	94.2 ± 18.4	0.12	89.8 ± 19.1	83.2 ± 19.0	94.2 ± 18.4	0.15	0.53
BMI, kg/m^2^	29.5 ± 5.2	31.7 ± 7.1	28.8 ± 4.0	0.17	31.2 ± 5.7	31.7 ± 7.3	30.9 ± 4.6	0.71	28.3 ± 4.4	29.6 ± 7.1	27.8 ± 3.2	0.28	**0.016**
**Comorbidities, *n* (%)**													
Any	68 (94)	19 (86)	49 (98)	**0.048**	28 (93)	10 (83)	18 (100)	0.08	40 (95)	9 (90)	31 (97)	0.39	0.73
Arterial hypertension	42 (58)	15 (68)	27 (54)	0.27	21 (70)	10 (83)	11 (61)	0.21	21 (50)	5 (50)	16 (50)	1	0.09
Respiratory disease	20 (28)	7 (32)	13 (26)	0.62	15 (50)	5 (42)	10 (56)	0.47	5 (12)	2 (20)	3 (9)	0.38	**<0.001**
Obstructive sleep apnea	6 (8)	2 (9)	4 (8)	0.88	4 (13)	0	4 (22)	0.08	2 (5)	2 (20)	0	**0.009**	0.2
Asthma	10 (14)	3 (14)	7 (14)	0.66	6 (20)	2 (17)	4 (22)	0.87	4 (10)	1 (10)	3 (9)	1	0.11
Other pulmonary diseases	6 (8)	3 (14)	3 (6)	0.29	6 (20)	3 (25)	3 (17)	0.59	0	0	0		**0.002**
Critical disease	21 (29)	6 (27)	15 (30)	0.82	8 (27)	2 (17)	6 (33)	0.33	13 (31)	4 (40)	9 (28)	0.49	0.7
Severe disease	51 (71)	16 (73)	35 (70)	0.82	22 (73)	12 (67)	10 (83)	0.33	29 (69)	6 (60)	23 (72)	0.49	0.7
Obesity (BMI ≥ 30 kg/m^2^)	27 (38)	10 (45)	17 (34)	0.36	17 (57)	7 (58)	10 (56)	0.89	10 (24)	3 (30)	7 (22)	0.61	**0.004**
Overweight (BMI ≥ 25 to <30 kg/m^2^)	32 (44)	6 (27)	26 (52)	0.05	9 (30)	2 (17)	7 (39)	0.21	23 (55)	4 (40)	19 (59)	0.29	**0.038**
Diabetes mellitus	19 (26)	4 (18)	15 (30)	0.30	10 (33)	3 (25)	7 (39)	0.45	9 (21)	1 (10)	8 (25)	0.33	0.27
Heart disease	16 (22)	4 (18)	12 (24)	0.59	7 (23)	2 (17)	5 (28)	0.5	9 (21)	2 (20)	7 (22)	0.9	0.85
Coronary heart disease	11 (15)	1 (5)	10 (20)	0.1	5 (17)	0	5 (28)	**0.047**	6 (14)	1 (10)	5 (16)	0.67	0.77
Atrial fibrillation	9 (13)	2 (9)	7 (14)	0.57	4 (13)	1 (8)	3 (17)	0.53	5 (12)	1 (10)	4 (13)	0.84	0.86
Chronic kidney disease	12 (17)	5 (23)	7 (14)	0.37	8 (27)	4 (33)	4 (22)	0.52	4 (10	1 (10)	3 (9)	0.96	0.06
Cancer	11 (15)	5 (23)	6 (12)	0.25	5 (17)	3 (25)	2 (11)	0.33	6 (14)	2 (20)	4 (13)	0.57	0.79
Ex-smoker	18 (25)	3 (14)	15 (31)	0.13	9 (31)	3 (25)	6 (35)	0.57	9 (21)	0	9 (28)	0.06	0.37
Current smoker	1 (10)	0	1 (2)	0.51	1 (3)	0	1 (6)	0.41	0	0	0		0.23
Cerebral arterial occlusive disease	2 (3)	0	2 (4)	0.35	1 (3)	0	1 (6)	0.42	1 (2)	0	1 (3)	0.58	0.81
Peripheral arterial occlusive disease	2 (3)	0	1 (2)	0.35	2 (7)	0	2 (11)	0.25	0	0	0		0.09
**Medical treatment**													
Steroids	70 (100)	22 (100)	49 (100)		30 (100)	12 (100)	18 (100)		42 (100)	10 (100)	32 (100)		
Antivirals	5 (7)	1 (5)	4 (8)	0.60	1 (3)	0	1 (6)	0.42	4 (10)	1 (10)	3 (9)	1	0.32
Monoclonal antibodies	2 (3)	2 (9)	0	**0.031**	0	0	0		2 (5)	2 (20)	0	**0.009**	0.23
Cytokine adsorption	1 (1)	0	1 (2)	0.51	1 (3)	0	1 (6)	0.42	0	0	0		0.24
**Lung function**													
TLC, L	6.4 ± 1.6	5.2 ± 0.8	6.9 ± 1.6	**<0.001**	5.7 ± 1.3	5.0 ± 0.9	6.2 ± 1.3	**0.009**	6.9 ± 1.7	5.5 ± 0.7	7.3 ± 1.7	**0.003**	**0.003**
TLC, % predicted	99.3 ± 17.5	103.4 ± 14.0	97.3 ± 18.8	0.19	96.2 ± 16.6	101.8 ± 16.8	92.5 ± 15.8	0.13	101.4 ± 17.9	105.3 ± 10.1	100.2 ± 19.7	0.45	0.21
VC, L	3.7 ± 1.1	2.8 ± 0.5	4.1 ± 1.0	**<0.001**	3.2 ± 0.9	2.6 ± 0.6	3.5 ± 1.0	**0.007**	4.1 ± 1.0	3.0 ± 0.3	4.4 ± 0.9	**<0.001**	**<0.001**
VC, % predicted	92.8 ± 15.5	96.6 ± 13.7	91.2 ± 16.1	0.17	89.4 ± 17.8	94.8 ± 16.7	85.8 ± 18.1	0.18	95.4 ± 13.2	98.9 ± 9.3	94.3 ± 14.2	0.34	0.11
VC < LLN, *n* (%)	23 (32)	6 (27)	17 (34)	0.58	13 (43)	4 (33)	9 (50)	0.38	10 (24)	2 (20)	8 (25)	0.75	0.08
VC < 70%, *n* (%)	7 (10)	1 (5)	6 (12)	0.33	5 (17)	1 (8)	4 (22)	0.33	2 (5)	0	2 (6)	0.43	0.1
RV, L	2.8 ± 1.1	2.3 ± 0.6	3.0 ± 1.2	**0.017**	2.6 ± 0.6	2.4 ± 0.5	2.7 ± 0.6	0.13	2.9 ± 1.4	2.2 ± 0.8	3.2 ± 1.5	0.06	0.18
RV, % predicted	117.7 ± 33.4	121.6 ± 27.0	116.0 ± 36.0	0.52	114.7 ± 27.7	123.3 ± 24.7	109.0 ± 27.1	0.16	119.9 ± 37.6	119.6 ± 30.7	120.0 ± 39.0	0.98	0.52
RV/TLC ratio, % predicted	112.0 ± 19.8	116.0 ± 13.3	110.2 ± 22.0	0.26	117.9 ± 20.7	118.7 ± 13.8	117.4 ± 24.7	0.87	107.7 ± 18.2	112.8 ± 12.6	106.1 ± 19.6	0.32	**0.030**
FEV_1_, L	2.9 ± 0.8	2.2 ± 0.5	3.1 ± 0.8	**<0.001**	2.4 ± 0.8	2.0 ± 0.5	2.7 ± 0.9	**0.021**	3.1 ± 0.7	2.4 ± 0.3	3.4 ± 0.6	**<0.001**	**<0.001**
FEV_1_, % predicted	90.3 ± 16.1	90.5 ± 15.4	89.7 ± 16.6	0.85	85.5 ± 19.4	88.2 ± 19.6	83.8 ± 19.6	0.55	93.2 ± 12.7	93.4 ± 8.5	93.1 ± 13.8	0.96	**0.047**
FEV_1_/FVC ratio, %	80.1 ± 6.7	80.9 ± 6.0	80.8 ± 7.1	0.94	81.1 ± 8.2	81.4 ± 7.7	80.8 ± 8.8	0.85	80.6 ± 5.5	80.3 ± 3.5	80.8 ± 6.1	0.83	0.8
FEV_1_ < 70% predicted, *n* (%)	3 (4)	1 (5)	2 (4)	0.92	2 (7)	1 (8)	1 (6)	0.78	1 (2)	0	1 (3)	0.58	0.38
Reff, kPa/(L/s)	0.3 ± 0.1	0.3 ± 0.1	0.2 ± 0.1	0.05	0.3 ± 0.1	0.3 ± 0.1	0.3 ± 0.1	0.38	0.2 ± 0.1	0.3 ± 0.1	0.2 ± 0.1	0.16	**0.031**
Reff, % predicted	82.8 ± 29.5	93.0 ± 28.7	78.2 ± 29.1	0.05	91.7 ± 31.3	97.8 ± 27.5	87.5 ± 33.8	0.39	76.4 ± 26.8	87.2 ± 30.4	73.0 ± 25.1	0.15	**0.030**
DLCO, mmol/(min*kPa)	6.1 ± 1.8	5.0 ± 1.2	6.6 ± 1.8	**<0.001**	5.1 ± 1.6	4.4 ± 1.1	5.6 ± 1.8	0.06	6.9 ± 1.6	5.7 ± 1.0	7.2 ± 1.6	**0.006**	**<0.001**
DLCO, % predicted	66.9 ± 14.4	64.4 ± 11.7	68.0 ± 15.5	0.34	60.2 ± 14.3	59.1 ± 11.3	61.0 ± 16.3	0.73	71.6 ± 12.7	70.7 ± 9.2	71.9 ± 13.7	0.8	**0.001**
z-score	0–2.01	0–1.97	0–2.02	0.89	0–2.60	0–2.55	0–2.64	0.73	0–1.58	0–1.29	0–1.67	0.31	**<0.001**
DLCO < LLN, *n* (%)	41 (57)	14 (64)	27 (54)	0.45	23 (77)	10 (83)	13 (72)	0.5	18 (43)	4 (40)	14 (44)	0.84	**0.004**
DLCO < 80% predicted, *n* (%)	56 (78)	21 (95)	35 (70)	**0.016**	28 (93)	12 (100)	16 (89)	0.25	28 (67)	9 (90)	19 (59)	0.08	**0.007**
DLCO/VA ratio, mmol/(min*kPa*L)	1.2 ± 0.2	1.2 ± 0.2	1.2 ± 0.2	0.89	1.2 ± 0.2	1.2 ± 0.2	1.2 ± 0.3	0.84	1.3 ± 0.2	1.3 ± 0.2	1.3 ± 0.2	0.6	**0.035**
DLCO/VA ratio, % predicted	85.2 ± 13.1	79.7 ± 11.0	87.6 ± 13.3	**0.018**	81.0 ± 12.8	76.5 ± 11.1	83.9 ± 13.3	0.12	88.2 ± 12.6	83.6 ± 10.0	89.7 ± 13.1	0.19	**0.019**
DLCO z-score	0–1.00	0–0.95	0–1.02	0.76	0–1.35	0–1.31	0–1.38	0.84	0–0.75	0–0.52	0–0.82	0.34	**0.006**
DLCO/VA ratio < LLN, *n* (%)	23 (32)	6 (27)	17 (34)	0.58	14 (47)	5 (42)	9 (50)	0.67	9 (21)	1 (10)	8 (25)	0.33	**0.023**
DLCO/VA ratio < 80% predicted, *n* (%)	28 (38)	9 (41)	19 (38)	0.82	16 (53)	6 (50)	10 (56)	0.78	12 (29)	3 (30)	9 (28)	0.91	**0.034**
Alveolar volume, L	5.0 ± 1.2	4.1 ± 0.6	5.4 ± 1.1	**<0.001**	4.5 ± 1.1	3.8 ± 0.6	4.9 ± 1.1	**0.006**	5.4 ± 1.0	4.4 ± 0.4	5.8 ± 1.0	**<0.001**	**<0.001**
6 min walk distance, m	473.6 ± 96.6	456.37 ± 74.0	481.1 ± 104.7	0.36	443.6 ± 116.3	439.6 ± 81.6	446.4 ± 138.6	0.9	489.7 ± 81.2	471.5 ± 67.0	495.6 ± 85.4	0.42	0.07
SpO_2_ after exercise, %	96.0 ± 2.2	96.5 ± 2.1	95.8 ± 2.3	0.29	95.3 ± 2.7	95.9 ± 2.8	94.9 ± 2.7	0.39	96.4 ± 1.9	97.0 ± 1.3	96.2 ± 2.0	0.25	0.05
Borg scale score													
Before exercise	0.9 ± 1.5	1.2 ± 2.0	0.8 ± 1.3	0.29	1.9 ± 1.9	2.1 ± 2.3	1.8 ± 1.6	0.65	0.2 ± 0.5	0.1 ± 0.2	0.2 ± 0.5	0.43	**<0.001**
After exercise	2.2 ± 2.4	3.2 ± 2.5	1.8 ± 2.2	**0.018**	4.4 ± 2.0	4.9 ± 1.9	4.1 ± 2.0	0.28	0.6 ± 0.8	1.1 ± 0.9	0.5 ± 0.7	**0.022**	**<0.001**
Pulmonary rehabilitation after hospital discharge, *n* (%)	8 (11)	2 (10)	6 (12)	0.77	5 (17)	1 (9)	4 (22)	0.38	3 (7)	1 (10)	2 (6)	0.7	0.19

Values are mean ± standard deviation, or number of patients (%). BMI, body mass index; DLCO, diffusion capacity of the lungs for carbon monoxide; FEV_1_, forced expiratory volume in 1 s; FVC, forced vital capacity; LLN, lower limit of normal; Reff, effective resistance; RV, residual volume; SpO_2_, oxygen saturation; TLC, total lung capacity; VA, alveolar volume; VC, vital capacity. Bold values indicate significant group differences (*p* < 0.05).

**Table 2:** Pulmonary function data at different timepoints after discharge from COVID-19-related hospitalization.

	Time after Discharge (*n* = 25)			
	6 Weeks	6 Months	12 Months	*p* Value *
TLC, L	5.58 ± 1.01	6.04 ± 0.93	6.03 ± 1.08	0.21
TLC, % predicted	89.51 ± 11.61	97.41 ± 11.64	98.03 ± 16.33	**0.049** ^§^
VC, L	3.42 ± 0.72	3.57 ± 0.73	3.48 ± 0.75	0.78
VC, % predicted	90.33 ± 12.24	95.16 ± 14.01	93.87 ± 14.72	0.42
VC < LLN, *n* (%)	11 (44)	6 (24)	7 (28)	0.29
RV, L	2.12 ± 0.52	2.48 ± 0.52	2.54 ± 0.73	0.06
RV, % predicted	97.28 ± 19.86	111.72 ± 21.91	114.94 ± 32.89	**0.039** ^§^
RV/TLC ratio	38.64 ± 6.4	41.14 ± 6.71	41.82 ± 9.67	0.32
RV/TLC ratio, % predicted	101.42 ± 12.73	108.54 ± 22.67	109.59 ± 28.26	0.37
FEV_1_, L	2.66 ± 0.57	2.75 ± 0.57	2.71 ± 0.62	0.86
FEV_1_, % predicted	90.34 ± 12.22	95.01 ± 16.88	93.3 ± 16.22	0.55
FEV_1_/FVC ratio, %	80.66 ± 7.66	80.86 ± 5.68	80.51 ± 4.86	0.98
Reff, kPa/(L/s)	0.28 ± 0.11	0.28 ± 0.1	0.28 ± 0.08	0.94
Reff, % predicted	92.17 ± 35.27	92.21 ± 34.27	94.74 ± 25.46	0.95
DLCO, mmol/(min*kPa)	5.64 ± 1.68	6.45 ± 1.49	6.16 ± 1.41	0.17
DLCO, % predicted	63.13 ± 13.49	72.80 ± 11.1	70.3 ± 11.13	**0.016** ^⧺^
DLCO < LLN, *n* (%)	18 (72)	10 (40)	12 (48)	0.06
DLCO < 80%, *n* (%)	22 (88)	20 (80)	23 (92)	0.46
DLCO/VA, mmol/(min*kPa*L)	1.19 ± 0.3	1.31 ± 0.24	1.28 ± 0.24	0.25
DLCO/VA, % predicted	83.01 ± 17.58	91.57 ± 12.19	90.32 ± 13.41	0.09
DLCO/VA ratio < LLN, *n* (%)	25 (100)	25 (100)	25 (100)	0.15
Borg score before exercise	0.69 ± 1.37	0.84 ± 1.12	0.66 ± 1.53	0.87
Borg score after exercise	1.94 ± 2.42	2.36 ± 1.98	1.83 ± 2.29	0.69

* Determined using analysis of variance. ^⧺^ Significant differences (*p* < 0.05) within the paired *t*-test between 6 weeks and 6 months if ANOVA is significantly different (*p* < 0.05). ^§^ Significant differences (*p* < 0.05) within the paired *t*-test between 6 weeks and 12 months if ANOVA is significantly different (*p* < 0.05) [in the paired *t*-test on TLC%, there was no significant difference between 6 w and 12 m]. DLCO, diffusion capacity of the lungs for carbon monoxide; FEV_1_, forced expiratory volume in 1 s; FVC, forced vital capacity, LLN, lower limit of normal; Reff, effective resistance; RV, residual volume; TLC, total lung capacity; VA, alveolar volume; VC, vital capacity. Data are presented as mean ± SD unless otherwise noted. Bold values indicate significant group differences (*p* < 0.05).

## Data Availability

The data that support the findings of this study are not publicly available due to privacy reasons but are available from the corresponding author upon reasonable request and with the permission of University Hospital RWTH Aachen.
